# Equity and capacity to benefit from early access to medicines schemes

**DOI:** 10.1186/s12939-025-02416-3

**Published:** 2025-04-10

**Authors:** Sarah J. L. Edwards, Paul Aliu, Joe Brierley, Søren Holm, Peter J. Pitts

**Affiliations:** Center for Medicine in the Public Interest, New York, NY 10024 USA

## Abstract

Many diseases, especially rare ones, have not gained the attention or support needed to attract dedicated research interest to be able to develop successful medicines. There is, thus, a significant unmet clinical need, not all of which is (or indeed can be) addressed through the evaluation of investigational treatments introduced within the confines of clinical trials. People with severe life-threatening conditions who are not eligible to participate in any ongoing clinical trials may be able to try investigational medicines through schemes facilitating early use of or expanded access to innovative medicines. Here, we focus on the issue of equity in such programmes. Standard metrics of clinical need which inform operational decisions about equity in resource allocation primarily rely on social goods which have already been evaluated, providing evidence to support the standard assessment of patient ‘capacity to benefit’ from given medical interventions. Notions of equity have only relatively recently been discussed within research and innovation generally and within the ethics of clinical trials in particular. Considerations of equity, however, require an overview of all these different patient pathways. We suggest that a new formal method to assess eligibility for early use of or expanded access to innovative medicines be used to capture both the severity of the condition and capacity for scientific or social value alongside clinical trials.

## Introduction

Many diseases, especially rare ones, have not gained the attention or support needed to attract dedicated research interest to be able to develop successful medicines. There is, thus, a significant unmet clinical need, not all of which is (or indeed can be) addressed through the evaluation of investigational treatments within the confines of clinical trials. People with severe life-threatening conditions who are not eligible for ongoing clinical trials may be able to try investigational medicines through early use or expanded access schemes, the most formalised of which are found in the United States of America (US), France and a handful of other countries [1]. These schemes include both an individual-named patient request made by the treating physician to the regulator or manufacturer (depending on the local regulations), and as part of a programme set up by the manufacturer to serve a cohort of similarly placed patients [2]. In some instances, these pathways have also provided access to an approved medicine for use in an unapproved indication.

This paper seeks to illuminate some of the ethical considerations largely understudied in current academic literature due perhaps to an initial reluctance to accept the premise behind early use of or expanded access to innovative medicines outside of the clinical trials environment, even perhaps surprisingly as a response to a public health emergency, considering that the US Food and Drug Administration (FDA) introduced such schemes in light of experience with patient groups during the Human Immunodeficiency Virus (HIV) crisis of the 1980s [3]. Such scepticism is now changing with growing awareness and acceptance of them, especially in rare diseases and in oncology [4]. It has been argued that, in most cases and in standard practice, any patient access to investigational products outside clinical trials would be inimical to medical science hence the common good, such that regulation should be yielded to restrict access for the purpose of maximising recruitment rates to clinical trials [5]. However, with increasing regulatory acceptance and appraisal of real-world data with new initiatives supporting its use [6], and recognition by companies that patients who are ineligible for clinical trials could nevertheless provide useful research data [7], there is growing interest in early access schemes alongside clinical trials where possible to provide complementary data.

In some instances, these data have supported the regulatory filing to permit marketing.[8] Example However, such programmes bring new ethical issues, including questions of appropriate thresholds of possible benefits and burdens, the possibility of genuine consent without so-called ‘false promise’ of benefit, and the (in)ability of treating physicians to aggregate and share data from individual patients easily. Here, we focus on the issue of equity in such programmes. Standard metrics of clinical need which inform operational decisions about equity in resource allocation primarily rely on social goods which have already been evaluated, providing evidence to support the standard assessment of patient capacity to benefit’ from medical interventions. Notions of equity have only relatively recently been discussed within research and innovation generally and within the ethics of clinical trials in particular. Considerations of equity require an overview of all these different patient pathways. We suggest that a new formal method to assess eligibility for early use of or expanded access to innovative medicines be used to capture both the severity of the condition and capacity for scientific or social value alongside clinical trials.

## Equity considerations

Equity refers to the concept of fairness and justice in various contexts, such as economics, law, and social issues [9]. In general, equity aims to ensure that all individuals have equal opportunities, rights, and access to resources or social goods, regardless of their background or circumstances. It involves distributing resources, benefits, and burdens in a way that promotes equality and addresses historical and systemic inequalities. Equity recognises that different individuals may require different levels of support to achieve similar therapeutic outcomes (‘healthcare equality’).

The debate over what counts as a just allocation of resources is often raised in the context of healthcare, where the safety and efficacy of technologies are known and are classed as social goods, yet there is a need to prioritise distribution across populations and disease states. Before examining the case of equity and use of new health technologies, we first outline the ethical considerations where health technologies have already been evaluated and approved for use so clearly count as social goods. Several core concepts underpin the different philosophical theories of justice in these settings. Allocation of resources based on the notion of need often seeks to prioritise individuals with the greatest need, thus allocating resources in proportion to the severity or urgency of the condition or situation. However, in traditional healthcare needs-assessment, those most likely to benefit from the available resources are regarded as most deserving. This conception thus aims to distribute resources based on the expected impact on health outcomes, considering the evidence of benefit and cost-effectiveness of interventions or treatments. It may involve weighing the anticipated benefits and costs to factoring in the severity of the condition to determine the most efficient use of resources at a population level, drawing on more consequentialist (or utilitarian) philosophical underpinnings than communitarian or solidaristic approaches.

It is widely acknowledged that, without strong policy initiatives, a free market tends to increase health inequalities, perhaps because of globalisation [10], although some analyses suggest that such inequality is not inevitable [11]. The relatively very worst off do not seem to be relatively better off, despite improvements in the average for most populations in the developed world as measured in absolute terms. In other words, they may be better off than they were but, when measured relative to the best off, there is a greater gap between them. Furthermore, such inequalities, especially those seen in global health, were amplified during the pandemic, creating a new impetus for examining policy initiatives to promote health equity [12]. However, measures of access to healthcare generally do not seem to emphasise equal opportunities, present an unclear picture of the causes of health inequalities and use a definition of need which relies on evidence of cost-effectiveness, helping to define those populations of patients who possess the capacity to benefit from them [13].

Under human rights frameworks, once a new medicine obtains a regulatory license to enter the market, there are certain individual entitlements under which it must be made available, while government funding policies and universal coverage schemes require allocation methods that are rational and factor in financial cost [14]. However, without evidence and critically a market license, it is less clear whether new technologies count as social goods at all and, if they do, how questions of equity might be considered. We now turn to how such questions have recently been raised in clinical research from where evidence of benefit is sought.

### Equity in research

The literature on ethics and research has also begun to grapple with ideas of equity in research, referring to ideas of fairness and inclusivity in research studies' design, conduct, and outcomes [15]. Indeed, the US National Academies recently published a report on the topic [16], while the European Commission has published new policy initiatives to promote greater equity in medical innovation [17]. Equity in research involves addressing disparities and disadvantages that certain groups face in accessing and participating in research, as well as the possible exploitation of some groups. To achieve equity in research, factors such as race, ethnicity, gender, socioeconomic status, disability, and other forms of diversity are considered important both morally and scientifically. This includes promoting diverse representation in research participants, research teams, and decision-making processes from early-stage development to post-approval access.

Equity in research also involves avoiding biases and stereotypes that may influence the research process and outcomes. Equity is thought to require recognising and addressing systemic barriers that prevent certain groups from participating in or benefiting from research opportunities. At the same time, the concept of health equity seeks to ensure that the risks and burdens of research do not fall disproportionately on certain groups while others reap the benefits. By prioritising equity in research, we ensure that the knowledge generated is representative, relevant, and applicable to all individuals and communities, leading to more inclusive and impactful scientific advancements regardless of geography or socioeconomic status.

However, those who participate in research are still not always representative of the specific population the research is intended to benefit. Until recently, many relevant groups were excluded from clinical research. For example, children, women, and ethnic minorities were historically excluded for various reasons. There are now regulatory requirements and incentives for researchers to gather evidence on these previously excluded groups sometimes before a new medicine is marketed so it can be suitably labelled and prescribed [18].

Before a market license has been granted, for many patients no established treatments exist, and the available evidence to support the use of investigational medicines may be thin and the biological rationale robust. To examine priorities for medical research, the World Health Organisation (WHO) uses another metric related to capacity to benefit or need, similarly based on consequential thinking, to gauge and then minimise the burden of disease’ or Disability Adjusted Life Years to address its potential social value [19]. It is also based on comparative assessments of disease states rather than prioritising those individuals who are worst off. Furthermore, research funding does not always follow the priorities to minimise the burden of disease.^20^

It is also worth stressing that almost all regulations require that clinical trials be prioritised within clinical research, and that early use of or expanded access to new medicines is only given when there is an available supply or when such a programme does not adversely impact any on-going clinical trial [20]. Yet considerations of equity can be complicated in such programmes partly because they are less selective than clinical trials, less closely monitored by professional supervision, with less data collected (if any data is collected at all). We first address the issue of cost and the rationale behind such programmes before turning to the issue of eligibility, which, if not considered carefully, could serve to compound existing inequities.

### Eligibility and expanding access

Early use of or expanded access to investigational medicines programmes are initiatives designed to provide patients with serious or life-threatening conditions the option of trying new drugs that regulatory authorities have not (yet) approved. These programmes are typically implemented when there is a high unmet medical need as defined by the severity of the condition and are based on risk/benefit assessments. These patients typically have no alternative treatment options available, including not meeting a clinical trial’s inclusion/exclusion criterion. The main purpose of the programmes is to provide these patients access to investigational drugs that show some promise in development or have a strong mechanistic rationale but have not yet been thoroughly tested or approved for market. Examples include various oncology indications, including Chronic Myeloid Leukaemia (CML), advanced breast cancer, advanced prostate cancer, advanced lung cancer, Duchenne’s Muscular Dystrophy, Alzheimer’s disease, and Amyotrophic Lateral Sclerosis (ALS) [21]. The number of expanded access programmes registered with clinicaltrials.gov has grown to over 900 since 2010 and now includes programmes for conditions such as glaucoma, depression, Mpox and Marburg [22].

Unlike access through clinical trials, there is increasing debate over support for early use of or expanded access to investigational medicines. Such access is often thought to be motivated by a sense of compassion (‘medical noblesse oblige’) or by the so-called ‘rule of rescue’, the psychological compulsion to help someone in great need [23]. In a typical scenario, the rule of rescue compels an agent to go above and beyond what would be considered cost-effective on a population level for an individual whose need is both immediate and life-threatening. There may be an automatic, intuitive reaction to help in such cases and be somehow symbolic for society. However, the rule of rescue does not by itself help us advance how we should think about equity of early access programmes once we have more people in need. Perhaps first come, first served as they are identified. A first come first served approach will, however, almost always lead to a situation where access is predominantly sought by knowledgeable patients with significant social capital.

Where individuals have different needs, vertical equity refers to different distribution proportionate to those needs. Where individuals are equally in need, yet there is not sufficient resource to treat both, there are issues of what is called ‘horizontal equity’ of resource allocation. Different allocation models have been considered in the past, especially when there are supply constraints or shortages [24].

What is less well appreciated is that early use of or expanded access to new medicines, when available, have ended up, in many cases, treating more patients across broader geographies than the related clinical trials on the compound. Examples include Imatinib, gefitinib, and erlotinib programs that treated 1000 s of patients. Expanded access to off label treatment to treat Mpox and Marburg outbreaks in Africa was also explicitly used to pave the way for clinical trials which would otherwise have been out of reach to patients.^16^ Placebo controlled trials are underway in the US funded by the National Institutes of Health (NIH) and in the UK through the National Institute for Health Research (NIHR).

Decisions over how much of the investigational medicine to dedicate to such a programme without compromising ongoing clinical trials are usually made early in the development process. However, they are influenced by several factors, including the nature of the specific drug, the size of the patient population, the country’s regulations, the policies of the drug manufacturer and the availability of the drug. If the investigational drug shows promising results, its availability in such programmes will be considered only when the patient has already tried the existing one(s) without success, i.e. when he has exhausted all other available treatment options. This approach is sometimes problematic for patients with serious or life-threatening conditions where existing treatments are known to have little or no efficacy in a similar population with a similar prognosis and time is of the essence. For example, some cancer patients are required to try several rounds of chemotherapy before trying novel immunotherapies despite knowing there is little chance of benefitting for them. There will be opportunity costs associated with exhausting options considered relatively futile.

Most importantly, for the doctors who facilitate access for patients, expanded access is mostly based on a realistic understanding of possible risks and benefits, however slim or unlikely, and personal hope [25]. Much has been made of the idea of giving ‘false’ hope, which we will not rehearse here except to suggest that, given the existence of early access programmes and clinical trials, they may present reasonable options for some patients despite the uncertainty.

Explicit criteria for assessing eligibility have already been attempted and seek to include some stab at including capacity to benefit.[26] Illuminating data also show that most expanded access programmes were in place within 6–12 months of the treatment obtaining FDA approval so that they will be offered on the basis of rather more than merely ‘promising’ data [27]. There will be some cases where the treatments are at a very early stage, but this is not the norm.

In some instances, the pathway for early use of expanded access to new medicines has also involved an approved medicine for use in an unapproved indication, as with the above use of antivirals for Mpox, in which case there will be more existing, and even direct, clinical evidence and safety data on which to base a judgement of likelihood of benefit and risk, even if limited to likely no harm. These programmes, more than most, will thus grapple with problems of horizontal inequities as there may be little to distinguish between the relative needs.

Some have even claimed that those most in need and from the lowest socioeconomic groups, known to be a social determinant of poor health, may be better served through a direct humanitarian approach, which simply prioritises their basic needs rather than comparing their relative outcomes with the best off.^14^ In this case, need should not be defined in a conventional way as capacity to benefit but should include some measure of *possible* benefit and *plausibility* along with accumulating data, a point to which we will return.

### Financial cost and social value

Perhaps an initial, yet sometimes mistaken, impression regarding who gets access to innovative healthcare is that those who can pay privately for treatment are more likely to get it [28]. In the US, current FDA guidance offers only the ability to recover direct costs, such as how much it costs per unit to manufacture the drug covering raw materials, labour, and non-reusable supplies and equipment needed to make the quantity of drug required for the patient’s use or costs to acquire the drug from another manufacturing source plus shipping and handling [29]. In short, drugs offered under an expanded access protocol cannot be priced for profit.

In most cases, the drug manufacturers provide the medication free of charge as part of the programme; however, ancillary costs may be associated with participation. The debate over the years has been over how hospital costs are covered, along with related assessments and follow-up. In some cases, the payor in some countries will not cover such associated costs if the treatment is not yet approved for market by the regulator. Patients participating in early access programmes may thus incur not inconsiderable out-of-pocket expenses related to the treatment, such as additional medical tests or monitoring required by the programme.

Insurance coverage for such programmes can also vary depending on the country and the specific insurance programmes. A comparison of the French Temporary Authorisation for Use (ATU), now Early Access program which covers costs, and the UK Early Access to Medicines Scheme (EAMS) which does not, suggests that the facility to charge has an unsurprising impact on which products are supported in the different jurisdictions [30]. Aside from the above mechanism in France, no insurance provider or payor covers the cost of an investigational drug itself. Some may cover the use of an approved drug in an unapproved indication but not a fully investigational drug that has never been approved in any indication, which represents the vast majority of early use/expanded access cases. Switzerland has recently sought to restrict its reimbursement policies on early access [31]. Payors in Switzerland and Austria will cover the use of an approved drug in an unapproved indication provided the patient is responding to the treatment after a 3–6-month period, mitigating the risk as opposed to not reimbursing it at all. Furthermore, many payors will not police off-label use strictly following the clinician’s therapeutic privilege so long as the drug is not expensive. Mandated insurance cover across the US for clinical trials has been attempted on several occasions but has not been widely endorsed by legislators, and expanded access programmes are not considered as part of cover and are not currently mainstream [32].

There is debate over the role of the commercial sector in expanded access programmes, which might seem commercially counterproductive or not worth the opportunity costs under current reimbursement policies.^30^ However, the introduction of right-to-try laws in the US has reignited concern over possible profiteering, for example, over the case of BrainStorm Cell Therapeutics’ considering a right-to-try strategy which crucially bypasses the regulator for expanded access to its experimental ALS compound that included charging a “semi-commercial” price significantly higher than its direct costs [33]. In a subsequent ironic step, the NIH took the unusual step of assuming responsibility for covering the costs of expanded access to some investigational drugs for ALS patients [34].

Without appealing to profit, the interests of the developer regarding expanded access may include a commitment to social or corporate responsibility in accepting a humanitarian approach to be the right thing and the opportunity to capture valuable data that help demonstrate effectiveness in populations outside those studied in clinical trials, potentially leading to broader indications. While data from real-world studies have already been included in applications for regulatory approval, there is less acknowledgement that greater post-market surveillance may be needed to corroborate or confirm ‘performance’ [35].

Equity of distribution may also call for using the outcome data and experiences so that others might learn as a condition of early or expanded access [36, 37]. While the mere chance of benefit could be enough to offset risks for the patients involved, reciprocal benefits could require that research is supported for the sake of other patients [38]. Here, we find the convergence of self-interest: more positive and predictive clinical outcomes, data points for value-based reimbursement, and a broader understanding of benefit/risk ratios.

Access to investigational treatments and adaptive regulatory programmes may also suggest that conversations about financial cost and evidence happen earlier than previously imagined under standard pathways, although this may ultimately reduce the cost for the investor [39]. A developer might now build early-stage payer conversations into its expanded access protocol to “pre-approve” reimbursement.

## Conclusion

The role of alternative therapies in determining eligibility can vary depending on the patient, the specific medicine, the country’s regulations, and the policies of the drug manufacturer. Ultimately, early access programs aim to provide potential benefits to patients with limited or no other treatment options. The availability and efficacy of alternative therapies are taken into account to ensure that early access to investigational drugs is offered to those most likely to benefit.

Equity in this specific context will always be tricky. In many cases, even within a particular health care system, due to contextual factors such as short supply treatment must occur at specialist centres. So, perhaps what can be achieved is not equity strictly speaking, but only distribution not biased by ethically objectionable factors. It is furthermore unclear whether equity concerns in this context should refer as much to horizon assessments between severely ill patients given caps of costs but to reciprocal benefit sharing so others might learn.

## Data Availability

No datasets were generated or analysed during the current study.
